# Non-surgical correction of gummy smile using temporary skeletal mini-screw anchorage devices: A systematic review

**DOI:** 10.4317/jced.58242

**Published:** 2021-07-01

**Authors:** Deema Alshammery, Nasser Alqhtani, Asmaa Alajmi, Lamis Dagriri, Nouf Alrukban, Rahaf Alshahrani, Shahad Alghamdi

**Affiliations:** 1 Department of Preventive Dental Science, College of Dentistry, Riyadh Elm University, Saudi Arabia; 2 Department of Oral and Maxillofacial Surgery and Diagnostic Sciences, College of Dentistry, Prince Sattam Bin Abdulaziz University, Al-Kharj 19942, Saudi Arabia; 3 Dental Students, College of Dentistry, Riyadh Elm University, Saudi Arabia

## Abstract

**Background:**

There are no studies that have systematically reviewed randomized control trials and/or prospective cohort studies that have assessed the significance of temporary skeletal mini-screw anchorage devices (TSAD) for the correction of gummy-smile. The aim of the present systematic review was to assess the significance of non-surgical correction of gummy smile using TSAD.

**Material and Methods:**

The addressed focused question was “Are temporary skeletal mini-screw anchorage devices effective for the correction of gummy smile?” Indexed databases were searched up to and including May 2020. Different combinations of the following key-indexing terms were used: anchorage; gummy smile, mini-screw; orthodontic; and vertical maxillary excess. The literature search was performed without time and language barriers. Randomized clinical trials and prospective cohort studies that addressed the focused questions were included. Risk of bias was assessed using the Downs and Black and Cochran collaboration tools. Letters to the Editor, commentaries, case-reports/series and articles published in non-indexed databases were excluded.

**Results:**

The initial search yielded 2118 studies out of which, four studies met the inclusion criteria and were processed for data extraction. All studies had a prospective research design. One study was a clinical trial and 3 had a non-randomized design. Results of the clinical trial showed no statistically significant difference in the extent of intrusion between the test- and control-groups. The non-randomized studies showed that TSAD are useful in reducing deep overbite. All studies had a high risk of bias.

**Conclusions:**

The TSAD are an effective and practical option in facilitating reduction of excessive gingival display or gummy-smile. However, further long-term follow-up, well-designed and power-adjusted clinical trials are warranted in this regard.

** Key words:**Anchorage, Excessive gingival display, Gummy-smile, Mini-screw.

## Introduction

An “ideal smile” requires the exposure of the entire length of the maxillary teeth with a gingival exposure of 1 to 3 mm (1). A state in which, the smile line moves in the apical direction beyond the anteroposterior teeth that results in an excessive exposure of maxillary gingivae during the smiling is termed as “gummy-smile” (2,3). Another definition for a gummy-smile is the exposure of at least 3 mm of the maxillary gingivae on smiling (1-3). Synonyms for gummy-smile encompass horse smile, high gingival smile line or high smile line (2). A gummy-smile is a common finding attribuTable to several intra- or extra-oral etiological conditions such as abnormal lip length or activity, gingival hyperplasia that reduces the length of the clinical crown, vertical maxillary excess (VME), or dentoalveolar extrusion (1,3). Excessive exposure of gingivae is an aesthetic concern especially among the youth (2,3); and it has been reported that a gummy-smile is more prevalent among females than males (2). For individuals presenting with a chief complaint of a gummy smile, it is essential to identify the cause of this aesthetic concern as it modulates the related treatment plan (1). Parameters that are assessed in this regard include evaluation of the oral hygiene and periodontal status, medical history (such as use of medications that may induce gingival hyperplasia), lip analysis (dynamic or static), dental analysis (incisal margin and crown length) and facial analysis (VME) (1,4).

Traditionally, invasive oral and maxillofacial surgical interventions are performed for the correction of VME (5-8). However, with recent advancements in clinical orthodontics and related research, it has been shown that temporary skeletal anchorage devices (TSAD) can successfully be used for aesthetic improvements among patients with a of gummy-smile (9-12). These studies (9-12) consider TSAD as a workable and rather conservative (non-surgical) mode of treatment for the management of gummy-smile compared with invasive surgical interventions (12,13). Ishida and Ono (13) corrected excessive overjet and a deep overbite with a bilateral Angle Class II molar relationship in a 36-year old female with a skeletal Class II gummy smile using TSAD. The treatment outcomes showed an improvement of gummy smile and lateral facial profile of the patient. The authors concluded that TSAD are effective in improving facial aesthetics in patients with a gummy-smile (13). It is however noteworthy that the positive outcomes reported by Ishida and Ono (13) were based upon evaluation of one patient. To the authors’ knowledge, there are no studies that have systematically reviewed and performed metanalysis on randomized control trials and prospective cohort studies that have assessed the significance of TSAD for the correction of gummy-smile.

With this background, the aim of the present systematic review was to assess the significance of non-surgical TSAD for the correction of gummy-smile.

## Material and Methods

-Ethics statement

The present study is a systematic review and the protocol is registered at the Riyadh Elm University Research Centre (IRB SRS/2020/10/194/183). There was no external source of funding for the present study.

-Focused question

The addressed focused question was “Are TSAD effective for the correction of gummy smile?”

-Inclusion and exclusion criteria

The inclusion criteria were as follows: (a) original studies; (b) clinical studies; (c) studies; (c) prospective studies; (d) randomized controlled clinical trials and (d) studies with follow-up. Letters to the Editor, retrospective studies, commentaries, case-reports and/or case-series, studies on animal models, in-vitro and ex-vivo studies; and articles published in non-indexed databases were excluded.

-Databases and search strategy

Indexed databases (MEDLINE, PubMed, Scopus, EMBASE, Google-Scholar, CINAHL, Cochrane Library, and LILACS, and the Saudi Digital Library) were searched up to and including May 2020. The literature search was independently performed by 2 authors (DA and NA). Different combinations of the following key-indexing terms were used: anchorage; gummy smile, mini-screw; orthodontic; and vertical maxillary excess. Any disagreements among the authors regarding study selection was resolved via discussion with a third Reviewer (AA). After reading the abstract and title of each relevant article, the full texts of the potential articles were also screened and assessed by two reviewers. The reviewers independently and blindly extracted outcomes and research characteristics by using the customized data extraction form.

-Protocol and registration

This systematic review and meta-analysis was conducted in accordance with the guidelines of the Preferred Reporting Items for Systematic Reviews and Meta-Analyses (14), and the Cochrane Guidelines for Systematic Reviews (15); and the protocol was registered in the International Prospective Register of Systematic Reviews (PROSPERO) (Registration # CRD192333)

Patients, Interventions, Control and Outcome

The Patients, Interventions, Control and Outcome (PICO) format was based on the following: (a) *P*=Patients with gummy-smile; (b) Intervention: management of management of gummy-smile using TSAD; Control= management of gummy-smile without TSAD or no treatment; (d) Outcome: improvement of gummy-smile

-Screening method, data extraction and risk of bias assessment

Information was synthesized by tabulating data according to (a) authors *et al*.; (b) study design; (c) number of participants; (d) gender of participants; (e) age of participants; (f) relevance of study characteristics in relation to gummy-smile; (g) relevance of study characteristics in relation to TSAD; (h) study outcomes; (i) power analysis; (j) amount of overbite reduction; and (k) conclusion. The Downs and Black tool for Assessing Risk of Bias(16) was used to assess the risk of bias of the included studies. The scale proposed by Downs & Black (1998) was used to rate the randomized and non-randomized clinical studies included. Scores of studies ranging ≥ 20, 15-19 and <14 were considered good, fair and poor, respectively (16). The risk of bias among randomized trials (RCTs) was evaluated using the Cochran risk of bias tool.(17) Every selected study was assessed with respect to the following aspects: (1) random sequence generation; (2) blinding of outcome assessors; (3) incomplete outcome data; (4) selective reporting; and (5) other bias. All RCTs were assigned an overall risk of bias, which was low if all domains showed low risk, high if more than one domain showed high risk, and uncertain if more than one domain considered to show an unclear risk. The authors (intra-examiner Kappa score: 0.82) of the present review assessed the risk of bias across the studies.

-Statistical analysis 

A statistical software (SPSS, Version 20, Chicago, IL, USA) was use to assess the Cohen’s kappa statistic that was used to measure the agreement among the reviewers.

## Results

-Outcomes of search strategy

**Figure 1:**
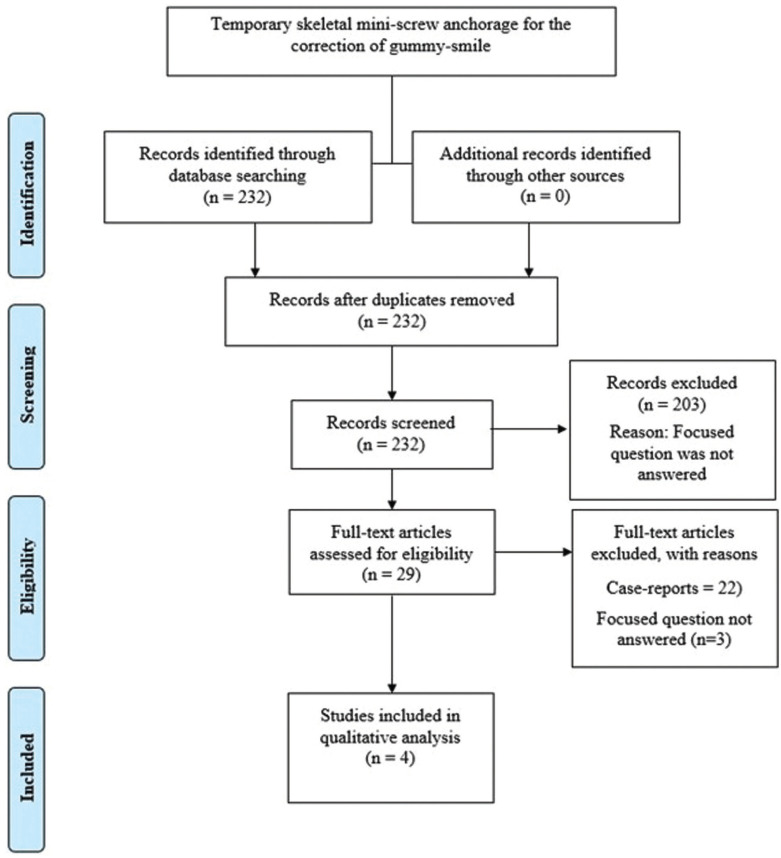
PRISMA flow diagram detailing the processing of the retrieved articles, from identification, screening, eligibility evaluation, and inclusion in the systematic review.

The initial exploration following electronic and manual searches yielded 232 studies. Studies, which did not abide by the eligibility criteria (n=203) were excluded at title and abstract level screening. From the remaining 29 studies, 25 articles were further excluded as they were either case-reports, review articles, retrospective studies, book reviews, case-series and/or conference abstracts were excluded. In total, 4 studies (18-21) were included and processed for data extraction (Fig. 1).

-Characteristics of the studies included

All studies (18-21) had a prospective research design. One study (18) was a clinical trial and 3 studies (19-21) had a non-randomized design. In the clinical trial(18), 30 patients (9 males and 21 females) were assessed; and in the remaining studies (19-21), the numbers of patients ranged between 10 and 16 individuals. In the study by Li *et al*. (19) all participants were females; and in studies by Polat-Ozsoy *et al*. (20) and Al-Falahi *et al*. (21), there were 3 males and 8 females and 9 females and 1 male, respectively. Two studies (19,20) did not report the mean age of the participants and in the remaining, individuals with ages ranging between 13 and 29 years were included (18,21). In all studies (18-21), two-dimensional lateral cephalograms were assessed. Prior sample-size estimation was performed in 1 (18) of the 4 studies (18-21) (Table 1).

**Table 1:**
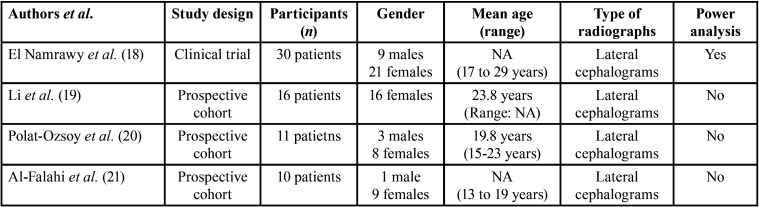
Characteristic of the included studies.

-Temporary anchorage device and orthodontic therapy related parameters

In all studies(18-21), patients with a deep bite were evaluated. Three studies(18,20,21) reported the severity of deep bite which ranged between at least 4 mm to up to 6 mm. The number of TSAD placed in the participants ranged between 10 and 30. Three studies(18,20,21) reported the intrusive forces applied to TSAD which ranged between 80 and 100 grams. Two non-randomized studies (20,21) reported the duration for which. TSAD were placed. These were 4.55 and 10.8 months (20,21). In the clinical trial (18), patients in the test-group (individuals that underwent intrusion of maxillary anterior teeth using TSAD) and control-group (individuals that underwent intrusion of maxillary anterior teeth without using TSAD) had TSAD inserted for 5.3 ± 1 and 4.8 ± 1 months, respectively. In the study by El Namrawy *et al*. (18), the rate of anterior intrusion among patients in the test- and control-groups was 2.6±0.8 and 2.9±0.8 mm, respectively. There was no statistically significant difference in the rate of anterior intrusion in the test- and control-groups (18). These results are summarized in Table 2.

**Table 2:**
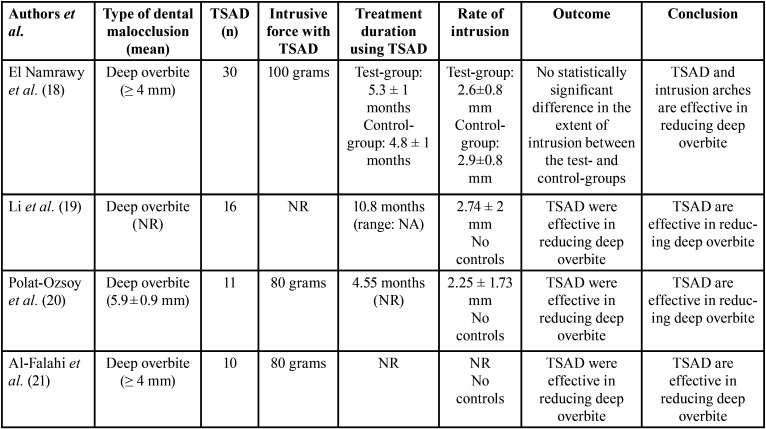
Temporary anchorage device and orthodontic therapy related parameters.

-Outcomes

Results of the clinical trial (18) showed no statistically significant difference in the extent of intrusion between the test- and control-groups. The non-randomized studies (19-21) showed that TSAD are useful in reducing deep overbite as shown in Table 2.

-Risk of bias

All non-randomized studies (19-21) had a high risk of bias. The risk of bias was high in the randomized trial (18) ( Table 3, Table 4).

**Table 3:**
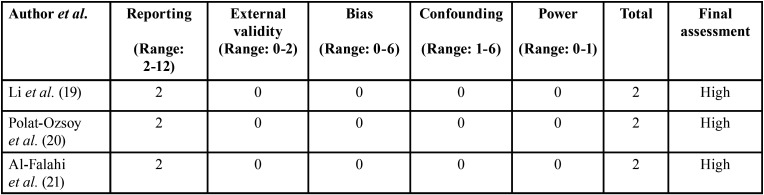
Risk of bias assessment of non-randomized studies using the Downs and Black scale (1998).

**Table 4:**
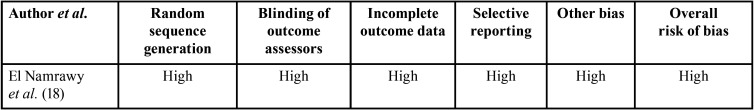
Risk of bias assessment of the clinical trial using the Cochran risk of bias tool.

## Discussion

The primary objective of the present systematic review was to evaluate the significance of non-surgical placement of TSAD for the correction of gummy-smile. Following an exhaustive literature search of various indexed databases, a limited number of clinical studies (18-21) addressed the focused question. In summary, 75% studies (19-21) had a non-randomized design; and the only clinical trial that encompassed test and control groups was performed by El Namrawy *et al*. (18). The authors initially intended to perform a meta-analysis on the studies (18-21) included; however, based upon the methodological inconsistency among the studies (18-21) such as duration of TSAD in function, age and gender of participants and absence of a control group in some studies did not allow a quantitative (meta-analysis) evaluation of the studies (18-21). Based upon such limitations, a systematic approach was adopted for the currently available evidence in the present study. In this regard, the authors suggest that further well-designed studies with a standardized methodology and longer follow-up periods are needed in this respect. Despite this limitation, it was interesting to note that 3 (19-21) out of the 4 studies (18-21) reported that TSAD are useful in the correction of deep bite. In this regard, it is tempting to postulate that TSAD can successfully be used for the correction of excessive gingival display, which is a common manifestation among patients with deep bite. However, the studies and conclusions reported in these studies (18-21) should be interpreted cautiously a number of factors may have influenced the reported results.

During the initial literature search, the authors identified numerous case-reports (12,13,22,23) that reported that the use of TSAD is a useful therapeutic strategy for a conservative (non-surgical) treatment of deep bite or gummy smile. However, it is demanding to generalize the results of case-reports and/or case-series as the reported conclusions are based on outcomes from a limited number of cases/patients. In clinical and experimental studies, power-analysis for sample-size determination (24) and blinding of the outcome assessors (25,26) are essential factors that minimize the risk of bias. Scrutiny of the included studies (18-21) showed that prior sample-size estimation was not performed in 75% of the studies (19-21) included. In this regard, the P-values reported in the included non-randomized studies (19-21) should be interpreted with caution. Although the results in the clinical trial by El Namrawy *et al*. (18) were power adjusted, the study had a high risk of bias. For instance, critical information related to parameters such as investigator blinding, allocation concealment (which might be challenging in such studies), and selective reporting remained unclear in this study(18) (Table 4). Similarly, all the non-randomized studies (19-21) also had a high risk of bias (Table 3). Therefore, the results of the included studies (18-21) remain debaTable and this is independent of the conclusions of the conclusions reported.

One aspect related to the methodology of the studies (19-21) assessed that may be criticized is that the authors used two-dimensional imaging technology (lateral cephalograms) for assessment of incisor intrusion using TSAD. With advancements in biomedical imaging sciences, the use of three-dimensional imaging technology such as cone beam computed tomography (CBCT) has increased in clinical orthodontics and related research (27,28). The CBCT imaging technology facilitates three-dimensional evaluation of anatomical entities including root angulation and morphology. However, utility of this advanced imaging technology in routine clinical orthodontic practice and research is still challenging due to obstacles such as expenditure, advance training and availability. It is worth mentioning that patients undergoing CBCT analysis are exposed to radiation to a significantly greater extent in contrast to conventional two-dimensional lateral cephalograms. In this regard, routine use of CBCT-based imaging is demanding from a bioethical standpoint (29-33).

A dilemma in clinical orthodontics and related research is the occurrence of orthodontically-induced inflammatory root resorption (OIRR). Studies (34-36) have shown that OIRR is also manifested following intrusion using TSAD. It is noteworthy that none of the studies included in the present systematic review addressed the occurrence of OIRR in the patient population. One explanation for this is the studies were primarily focused on intrusion using TSAD and most likely the possibility of long-term complications such as OIRR associated with incisor-intrusion using TSAD were disregarded. Further long-term-follow-up studies are needed to assess the OIRR following intrusion of anterior teeth using TSAD for the correction of gummy-smile.

## Conclusions

The TSAD are an effective and practical option in facilitating reduction of excessive gingival display or gummy-smile. However, further long-term follow-up, well-designed and power-adjusted clinical trials are warranted in this regard.
